# A Case of Amaurosis *Succesfully Treated by* Electricity

**Published:** 1804-02-01

**Authors:** Richard Smith

**Affiliations:** Chertsey, Surry, Member of the College of Surgeons


A CASE OF AMAUROSIS *successfully treated by* ELECTRICITY; *communicated by* MR. RICHARD SMITH, *of Chertsey, Surry, Member of the College of Surgeons.*

MRS. WILLISON, aged 37 years, by trade a straw hat maker, of a nervous irritable habit of body, afflicted likewise with dropsy of the ovarium, and labouring under complete ischuria for three years past, was attacked on the 12th of November last with a pain in the right eye, without any appearance of inflammation, (for some weeks previous to this she had found her eyes weak.) Conceiving it might arise from unusual exertion in her business by candle light, I recommended her to abstain from work a day or two, and use a slight vitriolic collyrium.

Nov. 14. Found herself incapable of elevating the eyelid, the pain had likewise affected the other eye; complained of a violent throbbing pain in the head, very different from a common head-ach she is sometimes troubled with, appearing to be, by her description, about the region of thalami. nerv. optic. the pulse slow and very full. In the evening she was incapable of raising her head from the pillow; both eyelids were now closed. On separating them, I found the pupils much dilated, and the retina insensible to the light of a candle placed close to the eye. Finding circumstances thus, I immediately took away ℥xij of blood from the arm, applied leeches to the temple, a blister to the crown of the head, and one behind the left ear; gave her calomel gr. ij. 4tis horis.

15. Found herself the same. Blisters having discharged well; I kept them open with cerat. canth. and gave her the following draught, as she was extremely low.

℞. Tinct. castor. et valer. ām. a ʒj. infus. cascarillæ ℥jss. f. haust 6ta. quaque hora sum cum pil. supra.

She pursued this plan without any apparent benefit until the 25th, when I was anxious to try the effect of electricity. Presuming the seat of disease to exist in the brain, I doubted the power of the electric aura to reach it; after a few sparks about the head and eyes, I directed some smart shocks through the brain, which gave, as I expected, much pain; I then left her until the following morning,

26. When I found the result to have exceeded my expectation. She had perceived a slight ray of light, and thought her head easier than the preceding day; in the evening I repeated the shock.

27. Found

27. Found her much better; could elevate the eyelids a little, and perceive light.

28. Much better; she now perceived large objects in the room, as the chairs, &c.

Dec. 1. Vision so far restored as to render the application of electricity unnecessary, as she could now perceive the smallest object, and was able to pursue her work; finds no inconvenience but from unusual exertion at her business, to which she is sometimes obliged to submit for the support of a young family.

How far the medicines might act in the above case, may be worth consideration; I think it fair to impute the success to electricity alone, since the other applications produced no beneficial effect until the shocks were made use of.

Electricity has been employed in similar cases without success; but as the manner of using it has generally been the slightest possible (i. e. the aura) I trust the event of this case may give encouragement for pursuing the more powerful remedy of the shock, which may be sent through the brain without apprehension of any unpleasant circumstance accruing from it, consequently may affect the origin of the optic nerve, usually the seat of this disease.

*January* 1, 1804.

